# Immediate effect of manual therapy techniques on the limitation of ankle dorsiflexion: a randomized, controlled, blind clinical trial protocol

**DOI:** 10.1186/s13063-021-05858-6

**Published:** 2021-12-06

**Authors:** Matheus de Castro Silva, Rodrigo de Marche Baldon, Carolina Lins, Gustavo Martins de Andrade, Gustavo Barros Braga de Castro, Lilian Ramiro Felicio

**Affiliations:** 1 Faculty of Physical Education and Physiotherapy, Pos Graduation Program UFTM/UFU, Uberlândia, Brazil; 2 Orthus Clinic Rehabilitation, Uberlândia, Brazil; 3 Faculty of Medical Sciences, Department de Orthopedics and Traumatology, UNICAMP, Campinas, Brazil; 4 Faculty of Physical Education and Physiotherapy, Federal University of Uberlândia, Rua Benjamin Constant, 1.286. B. Aparecida CEP: 38, Uberlândia, MG 38400-678 Brazil

**Keywords:** Dorsiflexion, Joint mobilization, Functional tests, Postural control

## Abstract

**Background:**

The range of motion (RoM) of dorsiflexion (DF) plays an important role in human mobility, such as absorption of body weight during gait deceleration, jump landings, balance, and eccentric movements. This limitation can generate potentially damaging movements. This way, evaluating techniques for DF RoM increase could help improve immediate performance in such functional activities. This being the case, the objective of this study will be to verify the sum effect of different joint mobilization techniques for DF gain in persons practicing physical activities and its relationship with functional performance and balance.

**Methodology:**

This is a randomized, controlled, and blind clinical trial. Fifty-four (54) volunteers will be recruited, aged between 18 and 40 years, who have DF limitations. After checking eligibility criteria, the participants will be submitted to a physiotherapeutic evaluation. A researcher, blind to evaluation and treatment, will perform the randomization of patients in groups: (A) Joint Mobilization - *Mulligan* Concept and (B) Joint Mobilization - *Maitland* Method. All volunteers will be submitted by two blind evaluators for randomization and treatment groups. They will realize the initial evaluation (A0), immediately after techniques (A1) and after 3–4 days of the technique application (A2). A different researcher, blind for evaluation, will perform the treatment, according to the randomization group.

**Discussion:**

It is already known that DF RoM limitation can lead to compensatory and potentially damaging lower limb movements and that joint mobilizations are effective to treatment. However, there is no consensus whether the application of these techniques would also improve aspects of dynamic postural balance and performance in individuals practicing physical activity, and whether the sum of two joint mobilization techniques could enhance this effect.

**Trial registration:**

Brazilian Registry of Clinical Trials (ReBEC) RBR-93xv9t. Registered on 09 April 2020.

**Supplementary Information:**

The online version contains supplementary material available at 10.1186/s13063-021-05858-6.

## Background

Ankle dorsiflexion (DF) range of motion (RoM) plays an important role in absorbing body weight during gait deceleration, jump landings, and eccentric movements [1]. The arthro-kinematic movement of DF is performed by the anterior sliding of the tibia over the talus, in closed kinetic chain (CKC) and, in open kinetic chain (OKC), the posterior sliding of the talus in relation to the tibia [2]. In this manner, the expected DF RoM for this ankle movement is 45° in CKC and 22° in OKC [3]. Some studies suggest that lower values may be considered inadequate and associated with patterns of potentially predictive movements of lower limb injury [2, 4].

These movement patterns are related to physical activities that require a wide amplitude of DF, such as jumping, step-up, and step-down, since the RoM of normal DF collaborates in the load absorption imposed on the lower limb [2, 5, 6]. In conditions of CKC activities, DF limitation could difficult progression of the tibia over the talus, limit knee flexion, decrease absorption capacity of eccentric loads and lead to compensatory knee and hip movements in the frontal plan e[7].

Moreover, although the predisposing factors to injury are not totally conclusive, some studies show that DF RoM deficit perpetuates ankle instability [8–11]. So, to evaluate interventions that promote this movement is of great relevance for choosing the best techniques or combining different techniques, especially of the joint mobilization to improve ankle RoM and, consequently, to reduce risk of injuries, to treat and minimize compensations and overloads in lower limbs.

Another aspect considered by Vallandingham et al. [12] is associated with DF RoM relationship and dynamic postural balance. The authors observed that DF RoM deficit reduces balance. This being so, evaluating postural control in physically active volunteers with ankle dorsiflexion deficit will bring important information for us to understand its association with functional aspects of postural balance.

Physiotherapy has tools that restore RoM such as joint mobilization, often used for this purpose [10, 13]. Specifically for the gain of DF, two techniques are known and have already been studied. Mobilization with Movement (MWM) of the Mulligan Concept [11, 13, 14] is the first of them, in which the author of the technique reports that it is performed with movement close to functional in CKC [14]. According to the systematic review by Weerasekara et al. [15], this could bring immediate benefits with its application for DF RoM. However, this same study shows that there is no consensus on immediate improvement regarding other outcomes [15].

Another technique of joint mobilization which is widely used for DF improvement is the anteroposterior passive mobilization (AP) of the talus in relation to the tibia of the Maitland Method [16, 17]. According to some authors, there is no superiority when comparing techniques for improving RoM [18, 19], but no studies were found that evaluated the additional effect on DF RoM, postural balance, and functional performance when associated with these two techniques of joint mobilization in the same treatment.

The hypothesis of the present study is that the sum of the techniques may bring additional effects on ankle DF range of motion compared to the isolated Mulligan Concept technique; therefore, this study aims to evaluate the immediate and short-term additional effect of joint mobilization in relation to RoM, functional performance, and postural balance in the physically active population.

### Primary objective

To evaluate the immediate and short-term effect of the associated joint mobilization techniques, Mulligan concept and Maitland method, for the ankle joint, on range of motion.

### Secondary objective

To evaluate the immediate and short-term effect of the associated joint mobilization techniques, Mulligan concept and Maitland method, for the ankle joint, on functional performance and balance*.*

## Subjects and methods

### Study design

**Fig. 1:**
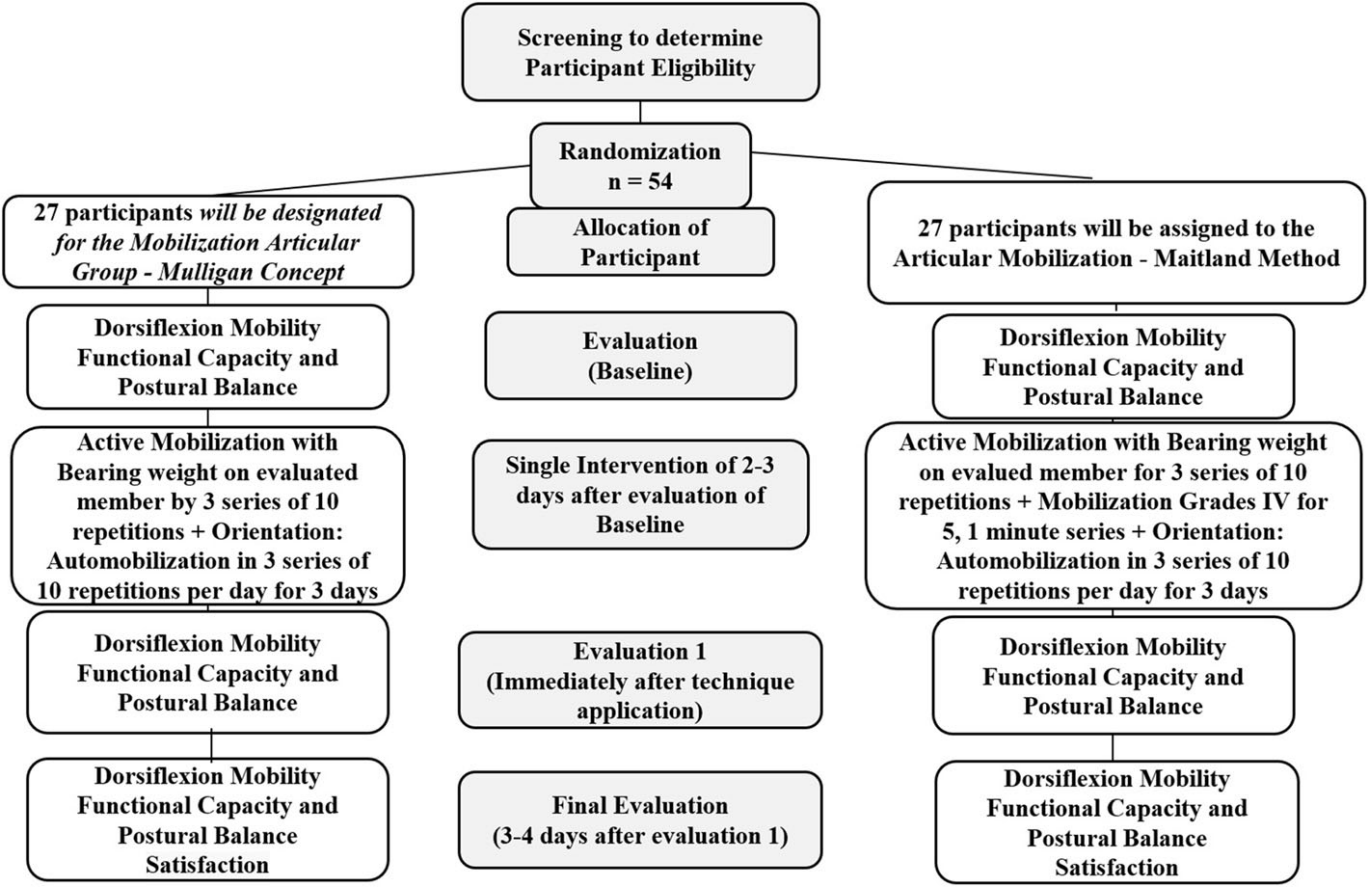
Flowchart of screening process and allocation of volunteers

This study is a randomized, controlled, blind clinical trial with two parallel groups (Fig. 1). The study was approved by the Human Rights Ethics Committee under protocol number CAAE: 30660520.1.0000.5152 and registered in the Brazilian Registry of Clinical Trials (ReBEC) (registration number: RBR-93xv9t). Recruitment will begin in September 2020 with a probable end date of November 22, 2021, as a result of the pandemic in the country*.* According to the flowchart (Fig. 1), evaluations will be conducted pre-intervention (A0), post-intervention (A1), and 3–4 days after intervention (A2). Variables to be observed are: Amplitude of Motion of Dorsiflexion of ankle in 20 closed kinetic chain and open kinetic chain, postural balance, and functional performance.

### Participants and evaluators

Sample calculation was performed using a difference of means and standard deviation, based on closed kinetic chain range of motion, of similar works, being RoM of DF in CKC, primary outcome, performed through the lunge test (LT), and minimum clinically relevant difference of 3.8 degrees [20], with an estimated standard deviation of 4.4°, and considering ANOVA statistical test of repeated measurements. The protocol considered a 0.05 alpha level and an 80% statistical power, being a sample size of 23 volunteers per group. A possible sample loss (15%) was computed, a total of 54 volunteers was considered for randomization.

Eligibility criteria are as follows: men and women between the ages of 18 and 40 will be recruited who have a range of motion (RoM) in closed kinetic chain of less than 40°, verified by lunge test. If the volunteer has bilateral ankle dorsiflexion limitation, the most affected side will be considered for analysis and intervention.

Exclusion criteria are as follows: presenting musculoskeletal injury in the lower limb, rheumatic diseases, cardiovascular diseases, dizziness, vertigo or changes in the vestibular system, neuropathic problems, or any other alteration that could harm the volunteer’s health or compromise performance of tests.

These individuals will be recruited through posters on the campus of the Federal University of Uberlândia (UFU) and by dissemination on social media and local media (TV and radio). So as to increase adherence, especially in the post-intervention evaluations, the volunteers will receive orientation, after final evaluation (A2), as to perform the exercises to maintain or improve ankle RoM.

Research will be developed at the Laboratory of Evaluation in Biomechanics and Neurosciences (LABiN) of the Faculty of Physical Education and Physical Therapy of the Federal University of Uberlândia, Minas Gerais, Brazil.

### Procedures

All participants will receive a Free and Informed Consent Term approved by the Human Rights Ethics Committee under protocol number CAAE: 30660520.1.0000.5152.

Volunteers will be submitted to individual physiotherapeutic evaluation containing ankle range of motion, history of injuries and previous treatments, physical activities performed, history of other diseases and, if necessary, special tests for hip, knee, and ankle to exclude musculoskeletal changes in lower limbs, these being the eligibility criteria. After all eligible volunteers will be randomized into two groups by a researcher not involved with evaluation, intervention, and recruitment of volunteers.

Volunteers will be informed that they will receive physiotherapeutic intervention, but will not know the difference between treatment groups or study hypothesis. Randomization will be carried out by Microsoft Excell® RAND command and placed in a brown sealed envelope, listed sequentially to hide participants’ allocation. Randomization will be performed in blocks of volunteers. These will be distributed in two groups, where the number “0” generated by the program will be considered as “intervention A” group and number “1” will be considered as “intervention B” group.

Two researchers that are not involved with randomization and treatment processes, will perform the evaluations to determine the outcomes. So, the evaluators will be blind for randomization and treatments applied. After, other physiotherapists (researchers), who will not be involved in evaluations and randomization processes, will be responsible for applying treatment techniques, according to the randomization group.

All outcomes data will be stored in secured folders and will remain with the evaluators, responsible for tabulating in Excell® spreadsheets after the end of data collection, thereby guaranteeing blinding.

The researcher involved with the processing of the biomechanical signals and functional tests will be independent, not knowing the previous steps. After processing the signals, the researcher responsible for the randomization will distribute the volunteers in the referred groups, for later statistical analysis, which will be performed by an independent professional.

### Intervention/control

Sixty-four volunteers will be randomly allocated in 2 groups: (A) Articular Mobilization - *Mulligan* Concept; (B) Articular Mobilization - *Mulligan* Concept + Articular Mobilization - Maitland Method. Both groups will be submitted to intervention according to the techniques mentioned.

**Fig. 2:**
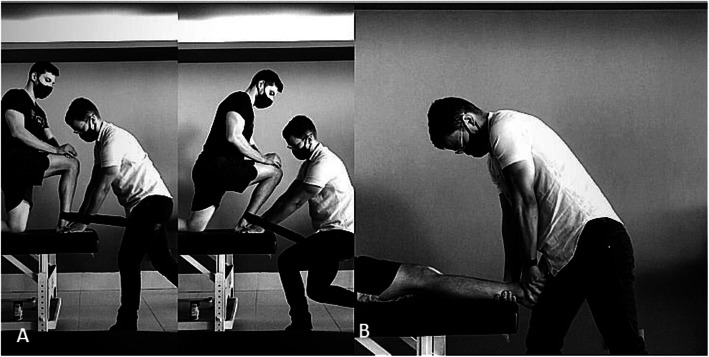
**A** *Mulligan* mobilization technique - Mobilization with Movement (MWM) and **B** Maitland method technique

Intervention group A will be submitted to *Mulligan* mobilization technique—Mobilization with Movement (MWM)—where the physiotherapist will manually stabilize with continuous anteroposterior (AP) direction pressure on the talus and a belt will be passed around the distal region of the leg region of the volunteer e and waist of the therapist, performing a posteroanterior (PA) pressure with the belt, in this way, the mobilization of the talus and the mobilization of the leg, should be maintained throughout the entire movement. The technique will be performed during ankle dorsiflexion in closed kinetic chain (CKC) (Fig. 2A), with the volunteer positioned in semi-kneeling position, and then performing active dorsiflexion with weight bearing, and at the end of the active movement*,* the patient will be guided to perform a final pressure called overpressure.

Intervention will be performed in 3 series of 10 repetitions. In addition, the volunteer will be guided to perform self-mobilization by performing oscillatory movements at the end of DF with CKC, in 3 series of 10 repetitions per day, aiming at gaining an ankle DF during 3 days [14, 19].

Intervention group B will also be submitted to technique cited in group A plus Maitland method technique, which will be applied after intervention A, in grade IV, with volunteer in dorsal decubitus, and foot out of stretcher. Mobilization will occur through a passive oscillatory pressure in AP direction in talus, for 5 series of 1 min [19, 21] (Fig. 2B). All techniques will be performed by a physiotherapist trained and experienced in clinical applicability of both techniques.

All stages of the clinical trial, randomization, blinding, and data collection are available to be audited by the institution’s Human Rights Ethics Committee, and the researchers are responsible for submitting six-monthly reports on the progress of the work.

### Outcome measurements

Three outcome measurements will be evaluated, 2–3 days before intervention application (A0), after immediate intervention (A1), and 3–4 days after intervention (A2).

#### Primary outcome

The primary outcome corresponds to DF ankle range of motion in CKC measured through lunge test (LT).

#### Secondary outcome

Secondary outcome corresponds to passive ankle DF measured in OKC by means of a goniometer, Postural Control which will be evaluated through the oscillation of CoP during the evaluation of Y balance test, which will be carried out on force platform, with functional performance evaluated through Y balance test (YBT) and triple hop test (THT) and, satisfaction evaluated through Medrisk satisfaction questionnaire [22].

Each result measurement is described below:

##### Range of motion

DF RoM will be measured in CKC performed by LT, this test being of high reliability for measurement of DF (ICC = 0.98) [9]. To this end, a smartphone and an application will be used for the acquisition of measurements (Clinometer^Ⓡ^), this application having already been validated [23].

**Fig. 3:**
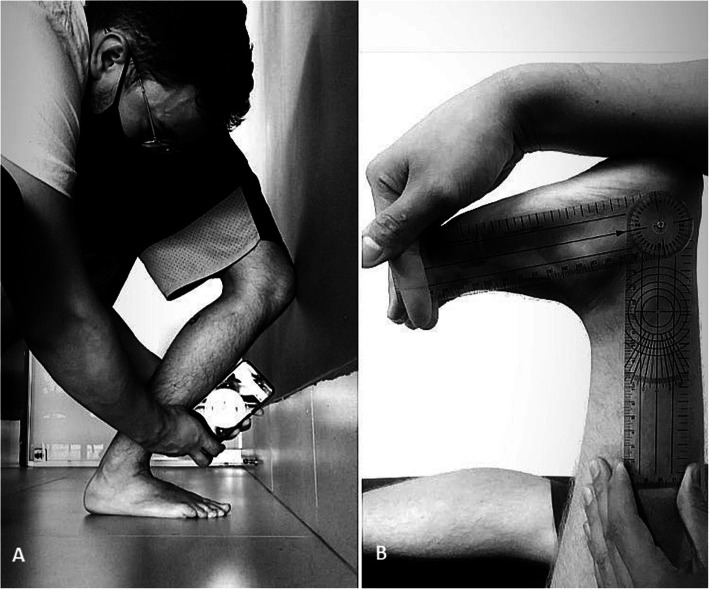
**A** Evaluation of ankle DF RoM during lunge test. **B** Evaluation of ankle DF RoM during goniometry

Lunge test will be performed with participant barefoot and with weight bearing on member evaluated (Fig. 3A). A 50-cm line will be fixed on the ground and a continuous 60-cm line will be demarcated on the wall to perform the test [3]. Cell phone device will be positioned 15 cm away from anterior tibial tuberosity [3]. Participants will be positioned with their feet along the line on the ground and then will be asked to move maximum DF, thereby bringing the knee closer to a vertical line drawn on the wall, without the foot moving from the ground (Fig. 3A). Once maximum DF is reached, the examiner will position the inclinometer on reference marked on the volunteer’s leg. The angle (in degrees) of DF and distance (in cm) from the tip of the 1st toe to the wall will be computed. The participant will return to the initial position. The procedure will be repeated 3 times and average calculated for later analysis [4].

RoM will also be measured in OKC by means of goniometry, a method validated by Petherick et al. in 1988 [24]. The participant will remain in a ventral decubitus position, knee positioned at 90°, and asked to perform ankle DF movement and then evaluator will help to complete maximum DF range passively. The goniometer will be positioned on the lateral malleolus with its central axis, fixed arm directed towards lateral epicondyle of knee, and mobile axis will be directed towards the 5th metatarsal (Fig. 3B). This procedure will be repeated 3 times and mean calculated for later analysis [24].

##### Postural Control

Postural control will be evaluated during the execution of YBT using the force platform.

Postural oscillation will be evaluated through analysis of the center of pressure (CoP) displacement with data obtained during the execution of YBT, performed during use of force platform (EMGSystem do Brasil® São José dos Campos, SP), with sampling frequency being 500 Hz. Force signals obtained (Fx, Fy, and Fz) and the moments of these forces (Mx, My and Mz), will be used to determine CoP oscillation, with the direction of CoP oscillation being considered, [+x]- anterior; [+y]- right and [+z]- upper directions will also be used.

Collecting environment will have controlled temperature and noise level minimizing interference in postural control. Participants will be previously oriented as to positions that should be performed during data collection. The volunteer must be initially positioned with face facing the posterior direction of platform (−Fy).

**Fig. 4:**
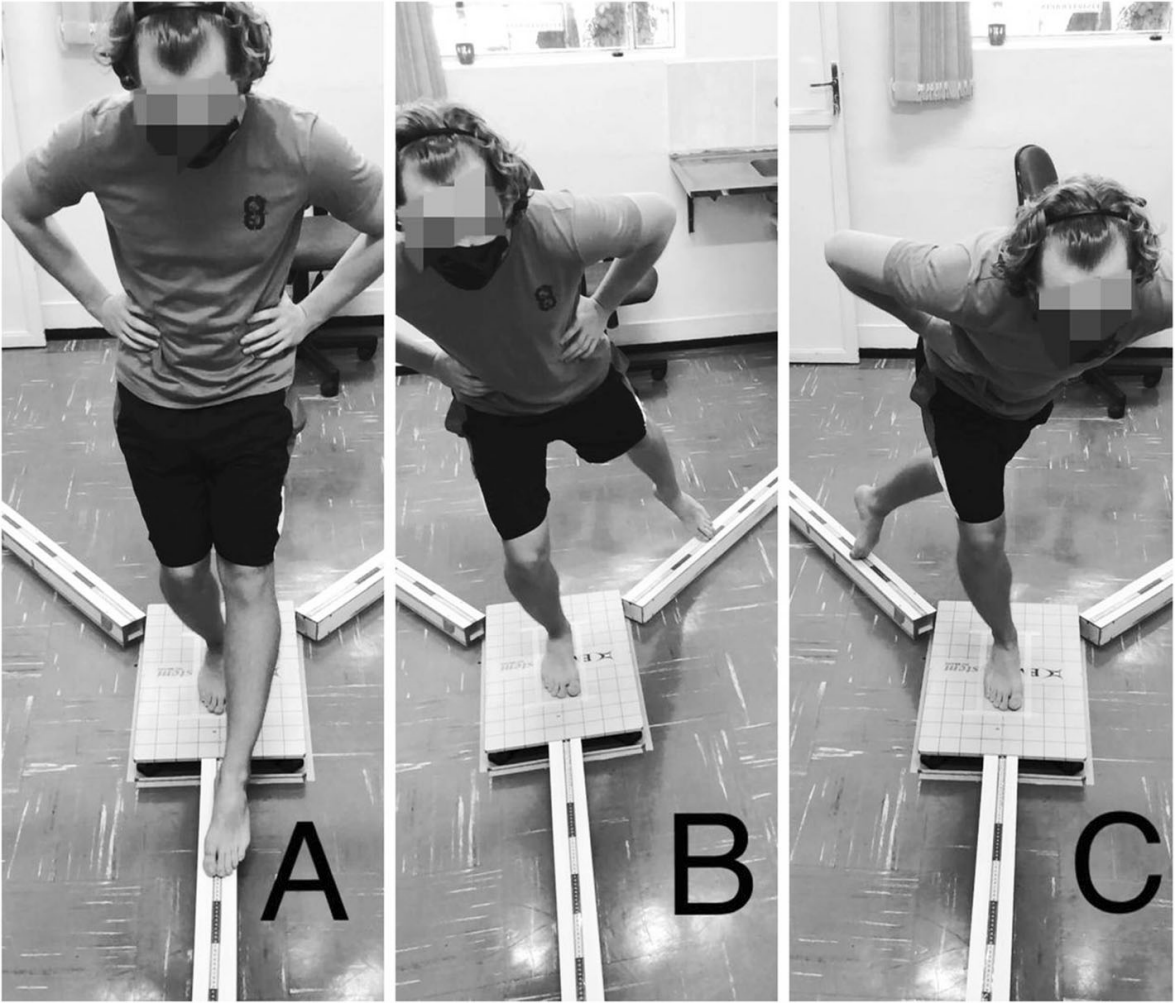
**A** YBT previous direction. **B** YBT postero-medial direction. **C** YBT postero-lateral direction

During the execution of YBT, the volunteer will be tested in *unipodal* support under the force platform. Participants will be positioned with their foot to be evaluated at intersections of lines, formed by previous, posterior-medial, and posterior-lateral directions marked on the force platform. Participants will be verbally instructed to perform maximum reaches in three directions, with contralateral limbs, with upper limbs positioned at waist level [25] (Fig. 4).

Participants will perform 3 familiarizations, followed by 3 tests for each direction. The test will be invalidated if hands do not remain at the waist area, if the position of support is not maintained, if the heel does not remain in contact with the platform during reach, if volunteer unloads body weight onto the contralateral limb, or if the participant loses balance during the test [25].

For kinetic analysis during YBT execution, data will be filtered using Butterworth 2nd order 2.5 Hz low-pass filter applied in direct and reverse direction. Variables will be calculated: average speed (cm/s); anteroposterior peak speed (Y) (cm/s); mid-lateral peak speed (X) (cm/s); anteroposterior displacement amplitude (Y) (cm); mid-lateral displacement amplitude (X) (cm); average displacement (XY) (cm); and reliable ellipse area (cm^2^) [26] for each test displacement direction.

##### Functional performance

The execution of YBT, carried out as described above, will also be computed for each direction, anterior, postero-medial, and postero-lateral, displacement, in centimeters, performed with contralateral limb to supporting member [25] (Fig. 4). Average displacement (AD) in each direction will be normalized using the length of support leg (LS) of the individual [25], and composite score (CS) will be calculated.

CS = AD (ant + postero-medial + postero-lateral)/3 × LS (×100)

To evaluate functional performance of THT, a demarcation will be carried out, consisting of a line 6 m long and 15 cm wide, perpendicular to a starting point [27].

**Fig. 5:**
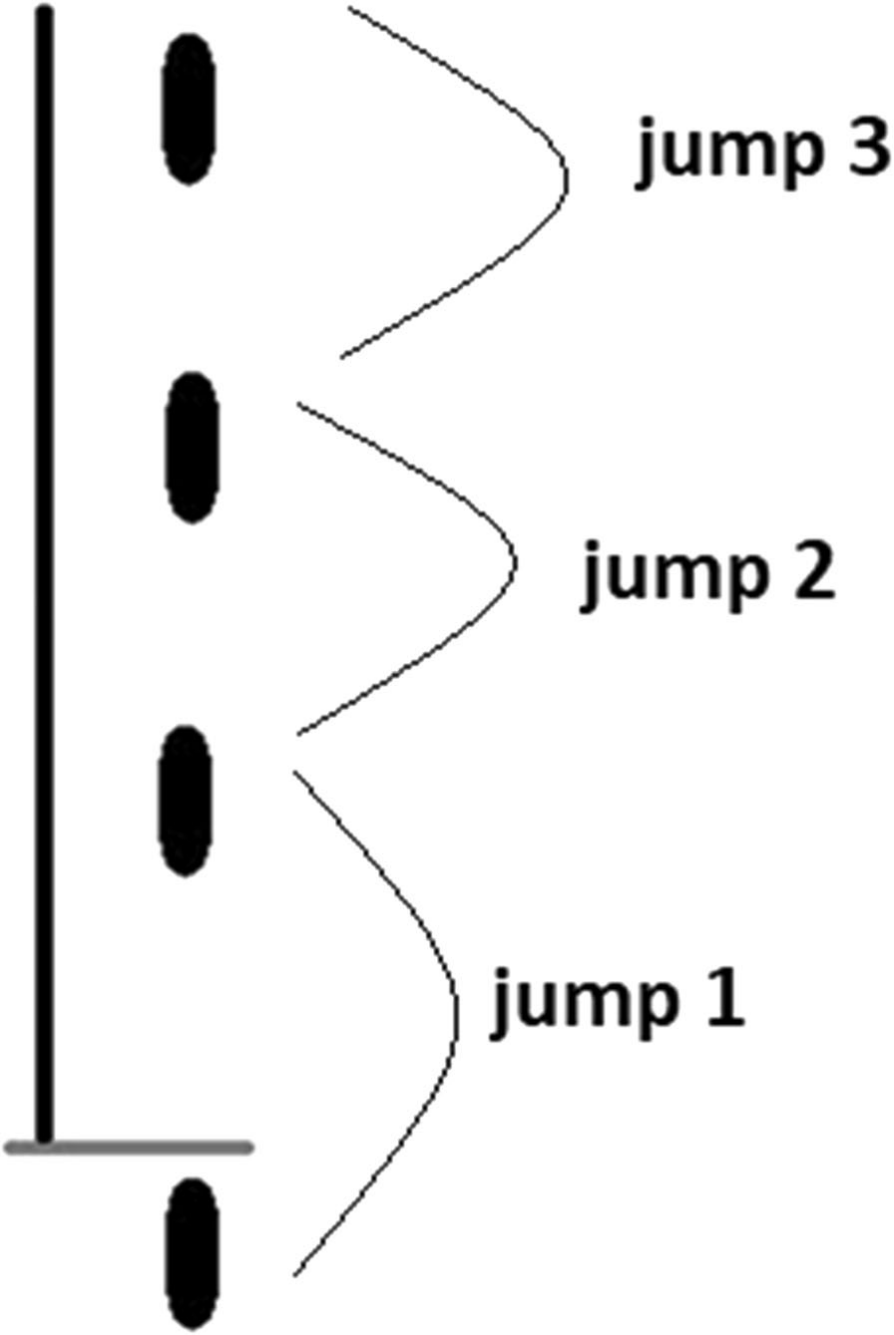
Triple hop for distance test scheme

This test is used to measure the combination of muscle strength, neuromuscular control, and ability to tolerate sports-related activities [28]. The participant must perform 3 maximum jumps with the same leg following the trajectory of the marked line. Subsequently, the distance between starting line and the tip of Hálux at the site of the last landing must be measured in centimeters [27] (Fig. 5). Three (3) repetitions will be carried out for the member to be tested, and the best result will be computed for later analysis.

##### Satisfaction

At the end of data collection after final reassessment (3–4 after applying technique), the Medrisk [9] satisfaction questionnaire will be presented, which is an instrument used to evaluate patient's satisfaction with physiotherapy treatment, composed of 13 questions with scores from 1 to 5 evaluating the quality of service and, 1 question evaluating one’s clinical perception of performance improvement.

### Statistical analysis

The normality test will be performed using the Kolmogorov Smirnov test. If null hypothesis is confirmed, comparison between groups will be performed using parametric tests, however, if null is not confirmed, non-parametric tests will be applied.

For parametric data, the effect of treatment will be evaluated by ANOVA two-way test for repeated measurements, considering a value of *p* ≤ 0.05. Data will be evaluated by intention to treat, through the imputation of the missing data using the *multipleimputation* method. For non-parametric data, the Kruskal-Wallis test will be used. All tests will be calculated using SPSS.

Clinical relevance of results will be confirmed by calculating the effect size (Cohen’s *d*) of significant differences found between assessments. The following effects will be considered: 0.00–0.49, small; 0.50–0.79, medium and above 0.80, large. Analysis of data will be made by intention to treat [29].

## Discussion

Literature shows that the difficulty of sliding the tibia over the talus can limit DF in CKC, which can limit knee flexion and decrease the ability to absorb eccentric loads [7]. This limitation may generate patterns of potentially predictive movements for lower limb injury [2, 4]. Some techniques and methods of joint mobilization are recognized for restoring DF RoM [10, 13]. However, there is still no consensus in literature on the addition of clinical effects on ankle DF RoM in the execution of these techniques, especially in the combination of the two best known techniques: *Mulligan* Concept and Maitland Method. In addition, no studies were found which assess maintenance of effect even if immediate or short term.

Although Vallandingham et al. [12] demonstrated an association between ankle DF RoM and dynamic postural balance, this factor is still not well established in literature. Understanding the influence of ankle RoM of DF on these variables could be an important parameter in understanding functional aspects, involvement in lower limb movement, and rehabilitation process.

## Trial status

“The trial record RBR-93xv9t, registered on April 9, 2020, had as its first evaluation date on January 20, 2021, with an expected completion date of data collection on November 22, 2021, and data were finalizing the analysis in March 2022.”

## Supplementary Information

**Additional file 1.**

## Data Availability

Not applicable.

## Abbreviations

RoM: Amplitude of movement
DF: Dorsiflection
OKC: Open kinetic chain
CKC: Closed kinetic chain
YBT: Y balance test
THT: Triple hop test
MWM: *Mobilization with Movement*
AP: Anteroposterior
ReBEC: Brazilian Registry of Clinical Trials
CAPES: Coordenação de Aperfeiçoamento de Pessoal de Nível Superior
LT: Lunge test
LaBiN: Laboratório de Avaliação em Biomecânica e Neurociências
