# Supplementary material for: Immediate effect of manual therapy techniques on the limitation of ankle dorsiflexion: a randomized, controlled, blind clinical trial protocol
Source: Trials. 2021 Dec 6;22:886. doi: 10.1186/s13063-021-05858-6 (PMC8647459; doi:10.1186/s13063-021-05858-6)
Supplement: Supplementary file 1 — Additional file 1. [file 13063_2021_5858_MOESM1_ESM.docx]

***Appendix 1:*** *Volunteer Trajectory and Timeline*

| **Volunteer Recruitment** | Volunteers will be recruited by means of posters on the campus of the Federal University of Uberlândia (UFU) and by dissemination in social networks and local media. |
| --- | --- |
| **Initial Evaluation (A0)** | All participants will receive an Informed Consent Form approved by the institution's Ethics in Human Rights Committee.  In the case of agreement, volunteers will undergo individual physical therapy evaluation to verify that they meet the eligibility criteria.  If the participant is included, he/she will undergo an evaluation containing the following outcomes: Functional performance, postural balance and range of motion in closed and open kinetic chain.  This initial assessment will have duration off approximately 40 minutes. |
| **Randomization** | After selection, all eligible volunteers will be randomized into two groups, (A) Joint Mobilization - Mulligan Concept and  (B) Joint Mobilization - Mulligan Concept + Joint Mobilization - Maitland Method. |
| 2 to 3 day interval | |
| **Intervention** | Volunteers will be submitted to the treatment techniques according to the group in which they were randomized. |
| **Reevaluation I (A1)** | Immediately after the Intervention, the participants will undergo a second assessment containing the following outcomes: Functional performance, postural balance and range of motion in closed and open kinetic chain.  This evaluation will have duration of approximately 40 minutes. |
| 3 to 4 day interval | |
| **Reevaluation II (A2)** | Volunteers will once more undergo an evaluation containing the following outcomes: Functional performance, postural balance, range of motion in closed and open kinetic chain, and treatment satisfaction.  This evaluation will have duration of approximately 40 minutes. |
